# Influence of the estimated glomerular filtration rate equation on carboplatin dosing: a real-world study

**DOI:** 10.3389/fphar.2025.1605458

**Published:** 2025-06-11

**Authors:** Ana Rita Fortunato, Cátia Barbosa, Ariana Araujo, Fernando Fernandez-Llimos

**Affiliations:** ^1^ Pharmacy Department, ULS Alto Ave, Guimarães, Portugal; ^2^ Laboratory of Pharmacology, Department of Drug Sciences, Faculty of Pharmacy, University of Porto, Porto, Portugal; ^3^ Applied Molecular Biosciences Unit (UCIBIO), Faculty of Pharmacy, University of Porto, Porto, Portugal

**Keywords:** carboplatin, drug-related side effects and adverse reactions, glomerular filtration rate, renal insufficiency, retrospective studies

## Abstract

**Background:**

Carboplatin is a renally excreted antineoplastic drug associated with myelotoxic effects. Doses are calculated according to the Calvert formula. The change from Cockcroft-Gault (CG) to the race-free Chronic Kidney Disease Epidemiology Collaboration (CKD-EPI) may have an impact on doses. The aim of the study was to evaluate the difference in carboplatin doses based on estimated glomerular filtration rate (eGFR) calculated using the two different equations (i.e., CG and CKD-EPI) applied to a real-world dataset of carboplatin administrations.

**Materials and methods:**

Retrospective study simulating the effect of switching to CKD-EPI on doses calculated using CG. Real-world data were collected on all carboplatin doses administered in a general hospital oncology day-care unit during 2023. Doses originally calculated using CG estimates were recalculated using CKD-EPI results. A Bland-Altman analysis was performed to assess the discrepancies between the two equations. Correlations with anthropometric data were examined.

**Result:**

A total of 487 cycles were administered to 126 patients with a mean age of 58.3 years (SD 12.6), 60.3% were female. There was a significant mean difference (p < 0.001) with a moderate effect (Cohen’s d = 0.474) between clearance calculated with CG and eGFR calculated with CKD-EPI. CKD-EPI calculated doses had a mean 52 mg higher (limits of agreement −107 + 211). Percentage differences between CKD-EPI and CG doses ranged from +70.9% (CG = 405 mg, CKD-EPI = 692 mg) to −24.3% (CG = 684 mg, CKD-EPI = 518 mg). Differences were strongly correlated with body mass index (BMI) (p < 0.001, R = 0.681).

**Conclusion:**

Clinically relevant differences were found between carboplatin doses calculated with CG and CKD-EPI. These differences were more relevant in male patients with low BMI.

## 1 Introduction

Carboplatin is an alkylating cytostatic drug that has been used since 1989 in various types of cancer. Since the first clinical trials, the hematologic toxicity of carboplatin is known (Colombo et al., 1989). Among the most commonly reported adverse events, myelotoxicity, nephrotoxicity, and peripheral neuropathy stand out (Rabik and Dolan, 2007; Carnovale et al., 2015).

Approximately 70% of carboplatin is excreted unchanged in the urine, so the dose must be adjusted to the patient’s renal function. Carboplatin clearance correlates linearly with GFR (Calvert et al., 1989). Renal function-adjusted carboplatin doses are usually calculated using the Calvert formula, which includes the patient’s target area under the curve (AUC) and glomerular filtration rate (Calvert et al., 1989). The appropriate dose is usually calculated at the start of treatment and modified if toxicity occurs. In addition, carboplatin doses should be recalculated if the patient’s clinical status changes or if the eGFR changes by more than 20% (Sandhu et al., 2022).

Despite the higher accuracy demonstrated by several existing methods to measure renal function using different laboratory tests (Chen et al., 2021), in clinical practice, and especially for the purpose of drug dose calculation, glomerular filtration rates (GFR) are estimated using different equations. Serum creatinine is commonly measured in hospital practice to identify acute kidney injury, very common in cancer patients (Oliveira et al., 2024; Janus and Desplanques, 2024). Traditionally, the Cockcroft-Gault (CG) equation has been used to estimate creatinine clearance (CrCl), which depends not only on renal function but also on factors such as muscle mass, diet, physical activity, and non-renal excretion (Cockcroft and Gault, 1976). This equation takes into account age, weight, and serum creatinine (SrCr) to estimate CrCl.

To avoid the influence of creatinine-related variability, including muscle mass fraction and tubular reabsorption (Fernandez-Prado et al., 2016), alternative equations for estimating GFR (eGFR) have been developed and validated against iothalamate. These equations, such as the Modification of Diet in Renal Disease (MDRD) (Levey et al., 1999) or the Chronic Kidney Disease Epidemiology Collaboration (CKD-EPI) (Levey et al., 2009), initially included ethnicity but not weight in their formulas. This race-based CKD-EPI was considered as preferred method to calculate doses in cancer patients (Janowitz et al., 2017), although other studies supported using weight- or BSA-adjusted CG (Samani et al., 2022). In 2021, the CKD-EPI equation was reformulated without ethnicity and validated against cystatin C^15^. A recent consensus between the National Kidney Foundation (NKF) and the American Society of Nephrology (ASN) recommended the use of the CKD-EPI without race (Delgado et al., 2022). Subsequently, pharmacists’ associations recommend the use of this equation to estimate doses in patients with impaired renal function (St Peter et al., 2024). Recent studies demonstrated the better performance of race-free CKD-EPI in multi-ethnic cancer populations, when compared to other CKD-EPI variations (Costa et al., 2023), but no sufficient evidence of comparisons between CG and race-free CKD-EPI exists for cancer patients (Schwenk, 2024).

Since a change in the eGFR equation used may affect the calculation of drug doses for patients (Castel-Branco et al., 2024), the objective of this study was to evaluate the difference in carboplatin doses based on eGFR calculated using the two different equations (i.e., CG and CKD-EPI) applied to a real-world dataset of carboplatin administrations.

## 2 Materials and methods

### 2.1 Study design

Retrospective study approved by the ULSAAve Ethics Committee (ref 90/2024). Based on Portuguese legislation (Law 21/2014), informed consent was waived due to the retrospective design of the study.

### 2.2 Data collection

Carboplatin-based treatments are administered in the outpatient oncology department of the ULSAAve general hospital. In July 2024, all carboplatin administrations in ULSAAve from January 1 to 31 December 2023 were retrieved from computerized patient records. For each patient, the following characteristics were collected: sex, weight, height, age, and SCr at the time of dose administration. To ensure anonymity, a unique code was assigned to each patient’s data by the clinical pharmacist in charge of oncology treatments.

### 2.3 Data analysis

Body surface areas (BSA) were calculated using the Mosteller equation (Mosteller, 1987):

$$BSA={weight}^{0.425}*{height}^{0.725}*0.007184$$

Body mass index (BMI) was calculated using the Quetelet equation (Quetelet, 1835):

$$BMI=\frac{weight}{{height}^{2}}$$

To calculate the ideal body weight (IBW), a BMI = 22 was considered to determine the corresponding weight.

CrCl was calculated using the CG equation (Cockcroft and Gault, 1976):

$$CrCl\left(mL/\min\right)=\frac{\left(140-age\left(years\right)\right)*weight\left(kg\right)}{72*SCr\left(mg/dL\right)}*A$$

A = 1 for males and A = 0.85 for females. Serum creatinine was obtained from patients’ medical records and had been measured by the hospital’s pathology laboratory using isotope dilution mass spectrometry (IDMS).

The 2021 CKD-EPI equation was used to determine eGFR (Inker et al., 2021):

$$eGFR\left(\text{mL}/\min/1.73m2\right)=142*\min{\left(\frac{SCr}{\kappa },1\right)}^{\alpha }*\max{\left(\frac{SCr}{\kappa },1\right)}^{-1.209}*0.993^{Age}*A$$

with κ = 0.7, α = −0.329 and A = 1.018 for females and κ = 0.9, α = −0.411 and A = 1 for males.

Carboplatin doses were calculated at each cycle using the Calvert equation with both renal function estimates (CG creatinine clearance and CKD-EPI eGFR) (Calvert et al., 1989):

$$Carboplatin\left(mg\right)=AUC*\left(CrCl+25\right)$$

No dose banding was applied to these calculations, but limits for the carboplatin doses (cap doses) are established at the hospital as follows: 300, 600, 750, and 900 for the desired AUCs of 2, 4, 5, and 6, respectively (Morrow et al., 2019).

Descriptive statistics were performed. Normality was calculated using the Kolmogorov-Smirnov (KS) test with additional visual inspection of the quintile-quintile (Q-Q) plot. Paired t-tests were used to compare the mean values of the two eGFR outcomes and the resulting carboplatin doses. Following the recommendations of the American Statistical Association (Wasserstein and Lazar, 2016), effect size measures (i.e., Cohen’s d) were obtained to supplement the null hypothesis tests. Pearson’s regression was used to calculate correlations between patient anthropometric measures and the percentage differences in the eGFR and the carboplatin doses obtained with the CKD-EPI compared to those obtained with the CG. Subgroup analyses by sex were conducted obtaining the equations of linear regressions for each sex. Multivariate linear regression analyses were conducted for the differences of carboplatin doses obtained with the two eGFR estimated including sex, CG dose, and BMI or BSA (independently to avoid collinearity). IBM SPSS v 28 was used, with significance set at p > 0.05.

Bland-Altman plots were generated to assess the agreement between the two methods for both eGFR calculation and carboplatin dose calculation based on the two eGFR results (Bland and Altman, 1986). To evaluate the discrepancy between the doses calculated by the two methods, the differences were expressed in absolute value and as a percentage of the mean eGFR. In Bland-Altman plots, the bias (i.e., the mean difference between the results obtained with the two methods) was plotted as a solid black line. The limits of agreement (i.e., the range within which 95% of the differences are expected to fall) were calculated as ±1.96 standard deviations from the mean difference. The regression line equation was calculated for the scatter plot of differences by mean eGFR (including the 95% confidence interval). Bland-Altman plots and related calculations were performed using R/RStudio (Posit, Boston, MS) with the packages BlandAltmanLeh (https://cran.r-project.org/web/packages/BlandAltmanLeh) and ggplot2 (https://cran.r-project.org/web/packages/ggplot2).

## 3 Result

A total of 126 patients with a mean age of 58.3 years (SD 12.6) and 60.3% females received 487 cycles of carboplatin during the study period. These patients had a mean body surface area of 1.68 m^2^ (SD 0.18), a BMI of 24.5 kg/m^2^ (SD 4.5), and an IBW of 57.9 kg (SD 5.3). Lung cancer was diagnosed in 61 patients (48.4%), breast cancer in 41 (32.5%), gynecological cancer in 17 (13.5%), and other neoplasms in 7 (5.6%).

During 2023, these 126 patients received 487 doses of carboplatin calculated with the CG equation, resulting in a mean dose of 527 mg (SD 162). Although the KS test was significant (p = 0.031), visual inspection of the Q-Q plot revealed minor deviations in the left tail (lower doses), which should not prevent us from using parametric tests (Sullivan and D'Agostino, 1992). The SrCr values used to calculate these doses had a mean of 0.78 mg/dL (SD 0.26), resulting in an eGFR of 84.3 mL/min (SD 28.3) using the CG equation.

**FIGURE 1:**
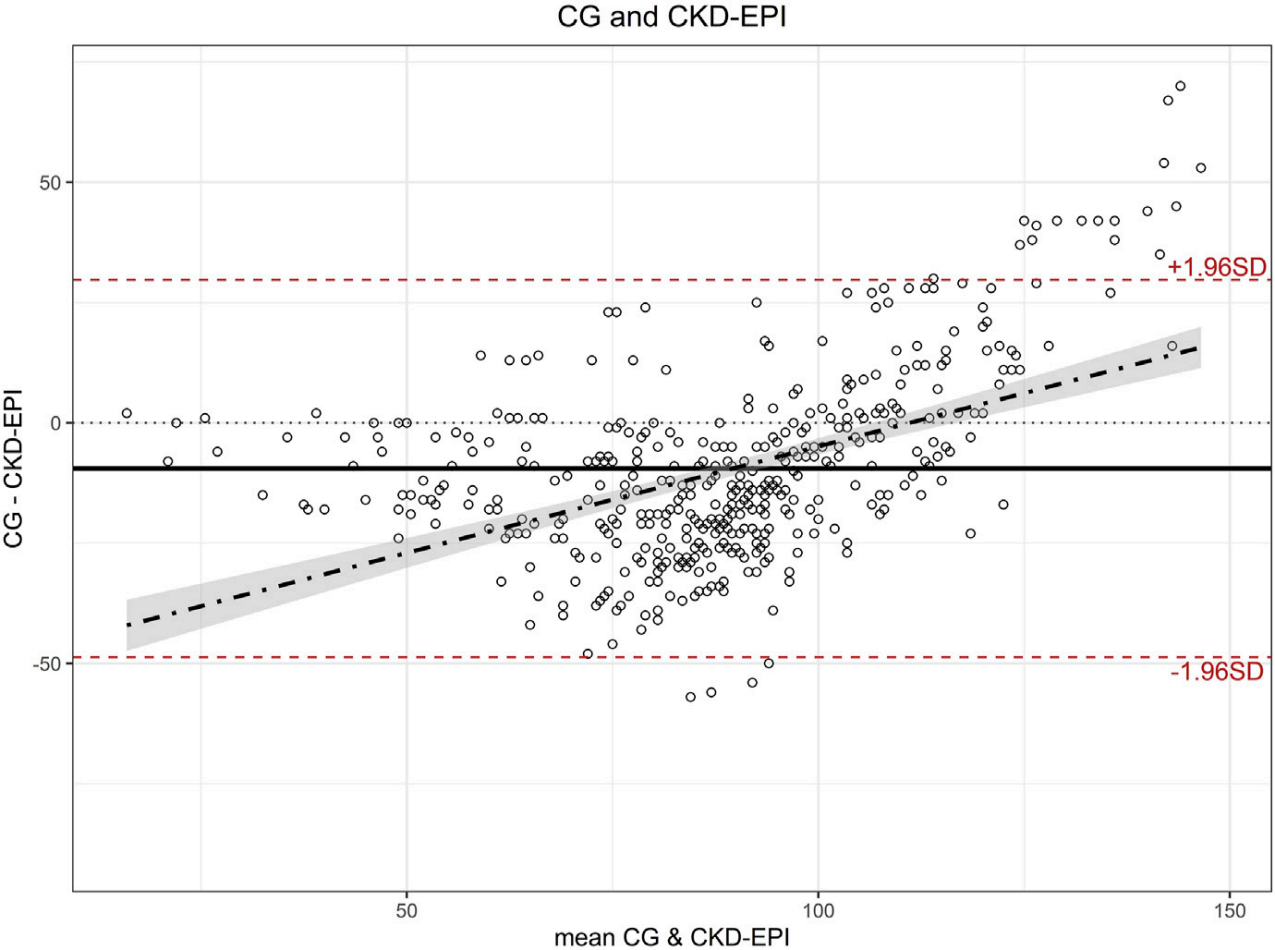
Bland-Altman plot for estimated glomerular filtration rate calculated with CG and CKD-EPI. CG: Cockcroft-Gault; CKD-EPI: Chronic Kidney Disease Epidemiology Collaboration.

When eGFR was calculated using the CKD-EPI equation, the mean eGFR was 93.8 mL/min/1.73 m^2^ (SD 18.7). A significant difference (p < 0.001) between eGFR calculated with both equations was observed in paired t-tests with a moderate effect size (Cohen’s d 0.474; 95%CI 0.380:0.568). The Bland-Altman plot of the discrepancies between the two eGFR results (Figure 1) showed a bias of −9.48, with limits of agreement of 29.7 and −48.7, and a regression line following the equation y = 0.47x−51.6, resulting in a null eGFR difference occurring at a mean eGFR of 110 mL/min.

**FIGURE 2:**
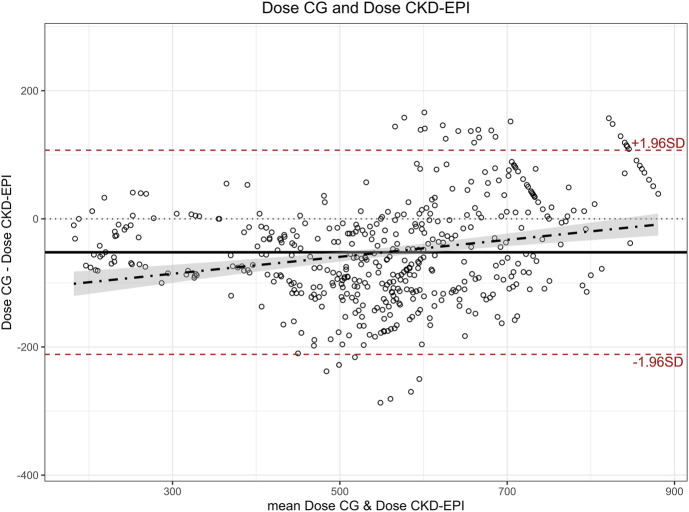
Bland-Altman plot for carboplatin dose calculated with CG and CKD-EPI. (Equation of the regression: y = 0.132x −125.3) CG: Cockcroft-Gault; CKD-EPI: Chronic Kidney Disease Epidemiology Collaboration.

**FIGURE 3:**
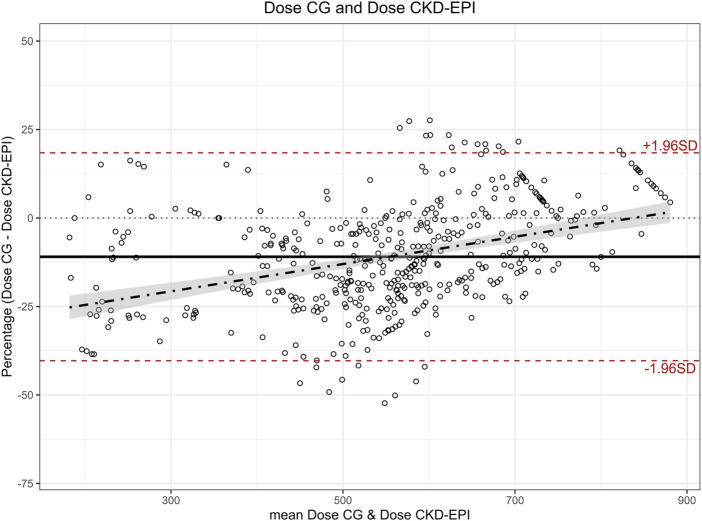
Bland-Altman plot for percentage differences in carboplatin doses calculated with estimated glomerular filtration rate calculated with CG and CKD-EPI. Note: Percentage was calculated as the difference CG - CKD-EPI over the mean dose of both methods. CG: Cockcroft-Gault; CKD-EPI: Chronic Kidney Disease Epidemiology Collaboration.

Mean dose calculated with eGFR estimated using CKD-EPI was 579 mg (SD 143 mg), which was 52 mg higher (SD 81 mg) than the mean dose calculated using CG (527 mg), representing an average increase of 12.9% (SD 17.1%). Paired t-tests showed a significant difference (p < 0.001) between the two doses with a moderate to large effect size (Cohen’s d 0.642; 95%CI 0.545:0.740). The Bland-Altman plot (Figure 2) showed a bias of −52, with limits of agreement of 107 and −211, and a regression line following the equation y = 0.132x−125.3. The Bland-Altman plot obtained using the percentage difference in doses calculated with both equations (Figure 3) showed a bias of −10.9%, with limits of agreement of 18.4% and −40.3%.

**FIGURE 4:**
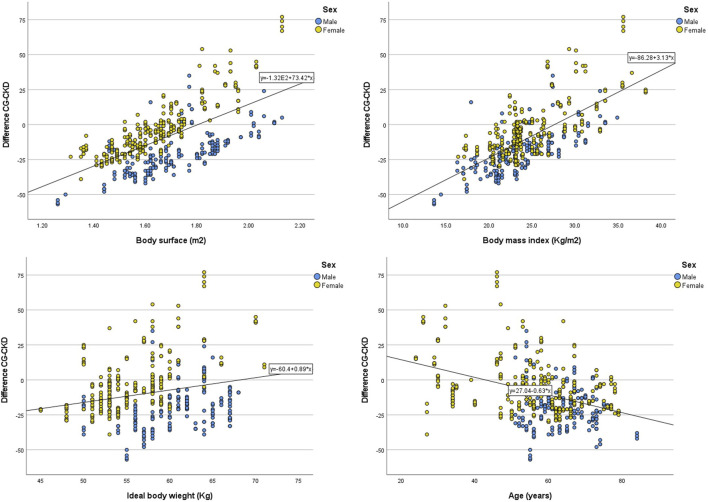
Correlation between patient characteristics and the difference of estimated glomerular calculated with CKD-EPI and CG. CG: Cockcroft-Gault; CKD-EPI: Chronic Kidney Disease Epidemiology Collaboration.

**FIGURE 5:**
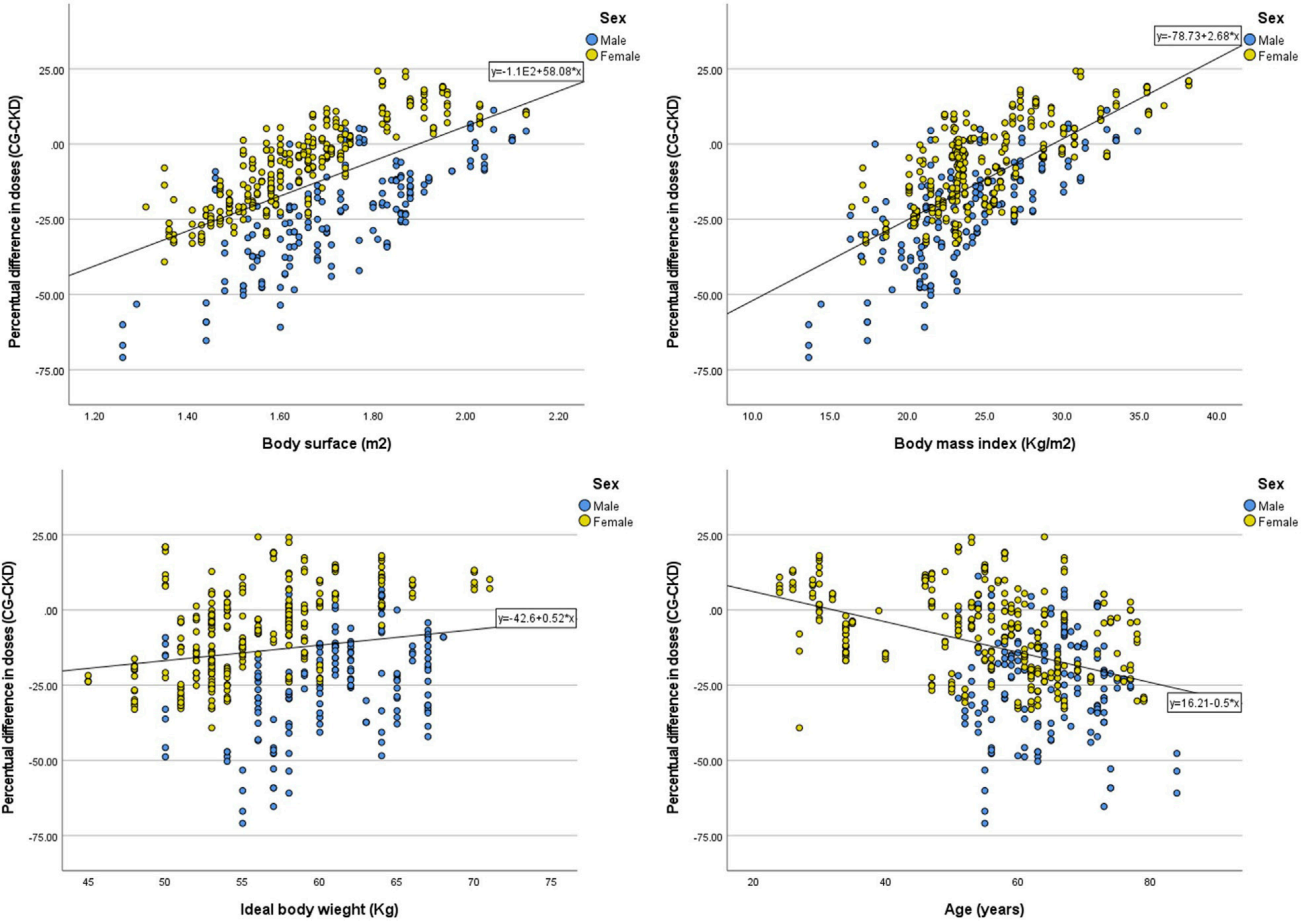
Correlation between patient characteristics and the difference in carboplatin doses calculated with CKD-EPI compared to doses calculated with CG. Note: Percentage was calculated as the difference CG - CKD-EPI over the CG calculated dose. CG: Cockcroft-Gault; CKD-EPI: Chronic Kidney Disease Epidemiology C.ollaboration.

A moderate to strong positive correlation was found between BSA and BMI with the difference in eGFR calculated with each equation (Table 1; Figure 4). Similar positive correlations were found with the percentage difference of the CKD-EPI dose compared to the CG-based dose. The most extreme values of these dose differences were +70.9% (CG dose = 405, CKD-EPI dose = 692) and −24.3% (CG dose = 684, CKD-EPI dose = 518). The percentage difference in carboplatin dose using CKD-EPI eGFR showed a moderate negative correlation with age and a weak correlation with IBW (Table 1; Figure 5). The subgroup analysis by sex showed that male patients presented greater differences than female patients in eGFR estimates and in carboplatin doses calculated with the two eGFR estimates (Supplementary Files 1, 2). No correlation was found for any of the variables with the value of SrCr.

**TABLE 1:** Correlation of the differences between results obtained with CG and CKD-EPI calculations and patient characteristics.

Patients’ characteristics	eGFR (CG) – eGFR (CKD-EPI)	Dose (FCG) – Dose (CKD-EPI)*
	Pearson’s R (p-value)	Pearson’s R (p-value)
Serum creatinine	−(0.494)	−(0.369)
Body surface area	0.641 (<0.001)	0.595 (<0.001)
Body mass index	0.677 (<0.001)	0.681 (<0.001)
Ideal body weight	0.232 (<0.001)	0.158 (<0.001)
Age	−0.412 (<0.001)	−0.385 (<0.001)

* Percentage over the dose (CG).

CG: Cockcroft-Gault; CKD-EPI: chronic kidney disease epidemiology collaboration.

The multivariate analyses showed strong associations of the percentage differences in doses calculated with the two eGFR estimates with sex, dose calculated with CG and BSA or BMI (Table 2).

**TABLE 2:** Multivariate linear regression analyses of the percentage differences in doses calculated with CG and CKD-EPI.

Covariates	B	95%CI	p-value	VIF
Sex	18.747	17.070:20.424	<0.001	1.192
Dose with CG	0.027	0.022:0.032	<0.001	1.235
Body surface area	62.136	57.503:66.768	<0.001	1.209
R-square = 0.766; F = 530.430; p < 0.001				
Sex	9.960	8.151:11.769	<0.001	1.081
Dose with CG	0.038	0.032:0.043	<0.001	1.140
Body mass index	2.130	1.929:2.330	<0.001	1.088
R-square = 0.701; F = 378.073; p > 0.001				

CG: Cockcroft-Gault; CKD-EPI: chronic kidney disease epidemiology collaboration; CI: confidence interval; VIF: variance inflation factor.

## 4 Discussion

Using a real-world cohort of patients, our study found significant and clinically relevant differences in carboplatin doses calculated using two different renal function equations (i.e., CG and CKD-EPI). The CG equation, which is traditionally used to estimate CrCl, resulted in lower doses than those obtained using the eGFR derived from the CKD-EPI (version 2021). Implementation of the CKD-EPI calculation will change carboplatin doses in an interval from +70% to −25% of those used with previous CG calculations, which should require extensive follow-up.

This study used a real-world cohort of patients receiving carboplatin treatments in a hospital day care department. This means that the study results are based on the actual characteristics of patients in the practice setting during the study period, which strengthens the external validity of the study. Although there was a wide variability in the anthropometric characteristics of the study patients, the study may not be generalizable to all patients in the world.

Another strength of the study is that the eGFR and carboplatin dose differences between the two equations used for dose calculation could be correlated with simple anthropometric characteristics of the patients. Thus, a risk-mitigation plan can be implemented, including an active pharmacovigilance program specifically targeted to these higher-risk patients (Silva et al., 2024).

Determining the appropriate dose of carboplatin has been a challenging task in patients with special anthropometric characteristics (Duffull and Robinson, 1997). For example, the Calvert formula based on CG-estimated CrCl resulted in overdosing in obese patients (De Jonge et al., 2002). Although these special patients could benefit from therapeutic drug monitoring, this practice is not usually implemented for regular carboplatin treatments and is reserved for special situations (e.g., high-dose treatments) (Moeung et al., 2017; Kicken et al., 2025).

A potential alternative to ensure the most appropriate dose of carboplatin could be the use of measured CrCl. Studies have shown significant differences between doses based on estimated and measured CrCl (Donahue et al., 2001). However, most hospitals continued to use estimated CrCl, mainly based on the CG equation. More recently, the original CKD EPI (the one that includes race variable) has been recommended because of its better performance (White-Koning et al., 2020; Tsang et al., 2021).

The NKF and ASN recommendation not to use eGFR equations with race variables (Delgado et al., 2022), supported by the AJHP (St Peter et al., 2024), may have implications for carboplatin dose calculations. In our study, the differences between the renal function estimates had a moderate effect size (d = 0.47), but the carboplatin doses calculated using both estimates had a moderate to large effect size difference (d = 0.64). On average, carboplatin doses calculated with CKD-EPI were 11% higher than those calculated with CG, but these differences were not equally distributed across patients. Male patients with low BMI had the largest differences between the doses calculated with the two equations.

The transition to the recommended race-free eGFR equations and the subsequent changes in the doses of carboplatin administered will require close monitoring of patient safety and efficacy. A first step in this risk minimization plan could be based on the implementation of therapeutic drug monitoring, especially for patients with low BMI. After implementation of the change, clinical pharmacists should pay special attention to myelosuppression in patients treated with carboplatin.

The Calvert formula also used the value of 25 as a constant representing the non-renal excretion of carboplatin (Calvert et al., 1989). This value was validated when CrCl was estimated using the CG equation. Further research should evaluate the potential modification of this value when using the non-renal CKD-EPI equation.

### 4.1 Limitations

This study has some limitations. It is a single center study with a small population. These two characteristics may limit the generalizability of the conclusion to other settings, but did not invalidate the results for the population attending the hospital’s outpatient cancer clinic.

## 5 Conclusion

Switching from the CG equation to the race-free CKD-EPI equation results in statistically significant and clinically relevant changes in carboplatin doses calculated using the Calvert formula. On average, CKD-EPI results in higher carboplatin doses that are more relevant in low-BMI male patients. To ensure patient safety and efficacy, a close follow-up plan should be established prior to implementing the equation switch.

## Data Availability

The datasets presented in this study can be found in online repositories. The names of the repository/repositories and accession number(s) can be found below: https://doi.org/10.17605/OSF.IO/8RFNC.
