# Supplementary material for: Influence of the estimated glomerular filtration rate equation on carboplatin dosing: a real-world study
Source: Front Pharmacol. 2025 Jun 11;16:1605458. doi: 10.3389/fphar.2025.1605458 (PMC12187738; doi:10.3389/fphar.2025.1605458)
Supplement: Supplementary file 1 [file Supplementaryfile1.docx]

**Supplementary file S1: Sub-analysis by sex of the between patient characteristics and the difference of estimated glomerular calculated with CKD-EPI and CG.**


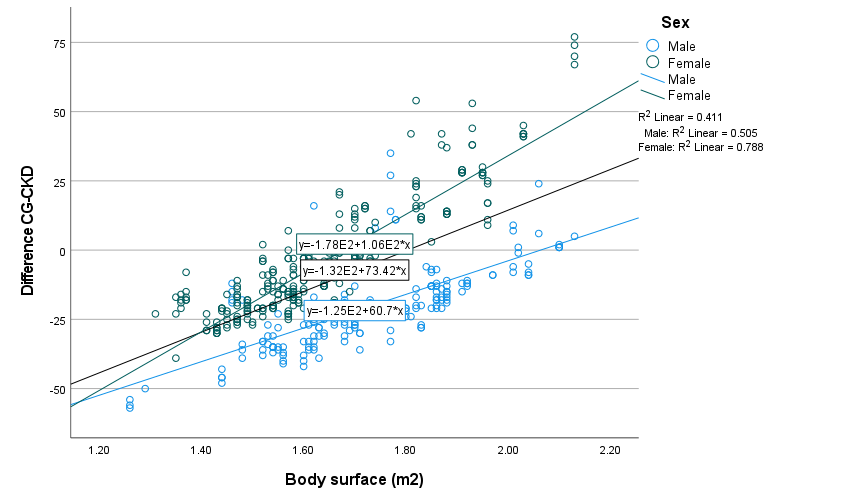

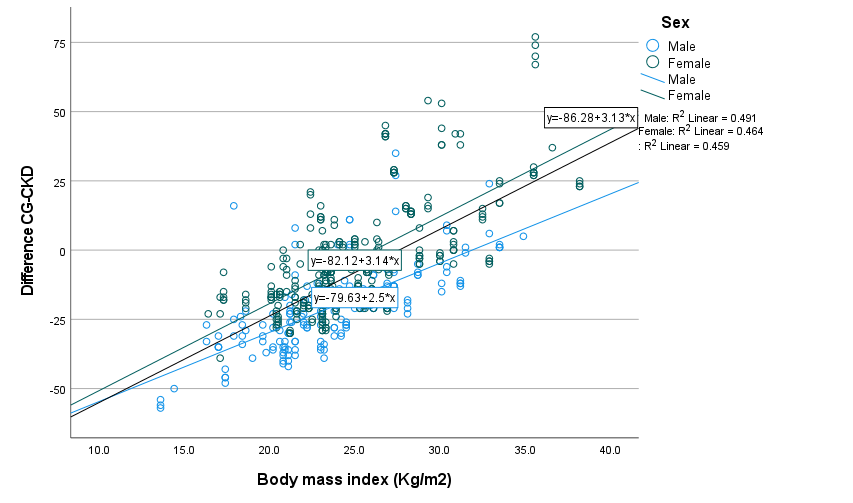


|  | Overall | Male | Female |
| --- | --- | --- | --- |
| Body surface | -132+73.42x | -125+60.7x | -178+106x |
| Bodi mass index | -86.28+3.13x | -79.63+2.5x | -82.12+3.14x |

**Supplementary file S2: Sub-analysis by sex of the correlation between patient characteristics and the difference in carboplatin doses calculated with CKD-EPI compared to doses calculated with CG.**


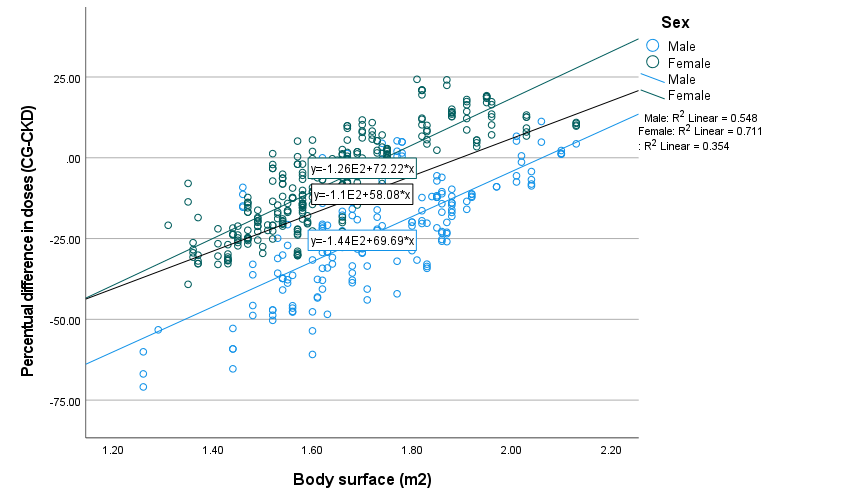

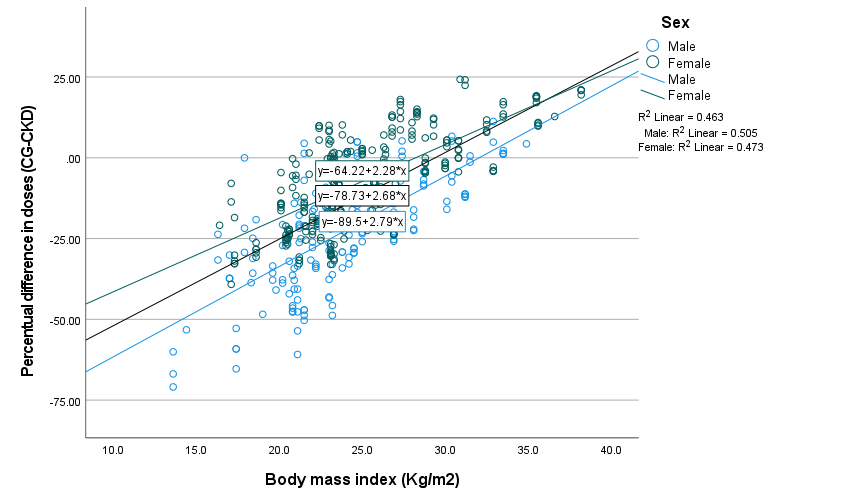


|  | Overall | Male | Female |
| --- | --- | --- | --- |
| Body surface | -110+58.08x | -144+69.69x | -126+72.22x |
| Bodi mass index | -78.73+2.68x | -89.5+2.79x | -64.22+2.28x |
